# Student and Educator Perspectives of Adapting to Remote Health Professions Education: A Mixed-Methods Study

**DOI:** 10.3389/fmed.2022.834228

**Published:** 2022-05-30

**Authors:** Mahbub Sarkar, Karen Liu, Arunaz Kumar, Dragan Ilic, Julia Morphet, Stephen Maloney, Elizabeth Davis, Claire Palermo

**Affiliations:** ^1^Monash Centre for Scholarship in Health Education, Faculty of Medicine, Nursing and Health Sciences, Monash University, Melbourne, VIC, Australia; ^2^Department of Obstetrics and Gynaecology, Faculty of Medicine, Nursing and Health Sciences, Monash University, Melbourne, VIC, Australia; ^3^Public Health and Preventive Medicine, Faculty of Medicine, Nursing and Health Sciences, Monash University, Melbourne, VIC, Australia; ^4^Nursing and Midwifery, Faculty of Medicine, Nursing and Health Sciences, Monash University, Melbourne, VIC, Australia; ^5^Primary and Allied Health Care, Faculty of Medicine, Nursing and Health Sciences, Monash University, Melbourne, VIC, Australia; ^6^School of Biomedical Sciences, Faculty of Medicine, Nursing and Health Sciences, Monash University, Melbourne, VIC, Australia

**Keywords:** adaptability, uncertainty, pandemic, remote education, health professions education

## Abstract

During the COVID-19 pandemic, universities across the world transitioned rapidly to remote education. Engaging with a curriculum that has been transitioned from in-person to remote education mode is likely to impact how students and educators adapt to the changes and uncertainties caused by the pandemic. There is limited knowledge about individual differences in students' and educators' adaptability to remote education in response to the pandemic. This paper explored healthcare students' and educators' adaptability experiences to remote education. Drawing on pragmatism, a convergent mixed-methods design was adopted. Data were collected between May and August in 2020 using an online survey, followed by interviews with students and educators of five large health courses at an Australian research-intensive University. Data included 476 surveys and seven focus group interviews with 26 students, and 95 surveys and 17 individual interviews with educators. Results were interpreted through an integration of quantitative and qualitative elements from student and educator experiences. Findings indicated that students were less adaptable than educators. Whilst remote learning was less appealing than in-person learning, some students adapted well to the new learning environment. Limited social learning, transmissive pedagogy, and lack of technical and non-technical skills were identified as factors that impacted upon the experience of students and educators. Navigating the challenges associated with remote education provided students and educators with a unique opportunity to improve adaptability—an attribute critical for future uncertainties in healthcare practice.

## Introduction

Adaptability refers to the cognitive, behavioral and emotional adjustments that individuals make to manage new, changing and uncertain circumstances, conditions and situations (1, 2). As Martin et al. (2) describes, cognitive adjustment refers to amending individual's thoughts, behavioral adjustment refers to amending actions, and emotional adjustment refers to amending affective responses, all to manage changing, new or uncertain events. A higher level of adaptability is associated with achievement, enjoyment, sense of purpose and life satisfaction (2). Adaptability is a key employability skill for success for graduates (3–6). There are uncertainties in the labor market caused by many factors, for example, technological advances, financial reforms and globalization (7, 8). Adaptability is thus required for graduates to cope with a rapidly changing, uncertain and highly competitive labor market (6).

Adaptability can be considered as a key requirement in all domains of work, life and education when natural disasters or global crises cause significant and lasting disruptions. March 2020, when the World Health Organization designated COVID-19 a global pandemic, marked a significant time in history when many things in our day-to-day lives were changed (9). The pandemic and the subsequent restrictions to physical gatherings interrupted conventional teaching and learning and disrupted the way tertiary education was delivered across the world. For health professions education, which has traditionally been supported through classroom-based teaching and work-integrated learning, the pandemic forced health professional courses to rapidly shift to a new mode of delivery. Pandemic-related restrictions considerably interrupted standard practices, requiring re-imaging of curricula, its delivery, and assessments (10, 11). Barriers included the loss of collaborative clinical experiences with peers, educators and patients, and the cancellation of face-to-face clinical workshops (12, 13). For preclinical medical education, whilst remote learning offered increased flexibility to students, these changes negatively affected the quality of instruction and student participation in learning (14).

The pandemic-related changes caused educators to adapt to different teaching philosophies and different modes of delivery (15). As Bao outlined, educators needed to adopt specific pedagogical strategies to maintain social learning for students in the online environment while achieving a smooth transition for them (16). Educators converted face-to-face teaching sessions to synchronous video conferenced lectures (e.g., using various video conference platforms) and asynchronous learning episodes. To promote student engagement in online learning, a range of online tools and remote teaching strategies were enacted (e.g., chat box, breakout rooms, polling, virtual whiteboards, annotate functions, quizzes and games) (17, 18). While many of these online tools were used pre-pandemic, their uptake was inconsistent, particularly with educators and students who had limited exposure to their implementation in the teaching and learning and learning context (15). A rapid response to the changes and preparation for remote learning left little time to adjust (19). Consideration was needed regarding logistics (e.g., technological devices, reliable network and a quiet study place), focus on learning remotely with limited academic advice, career guidance and mental health support (19). Additionally, both students and educators had to cope with the sense of isolation, stress and anxiety given the pandemic-related restrictions (20, 21). Understanding how online remote education influenced the preparation of health care professionals for practice is at the forefront of the global education agenda.

While there is a plethora of research exploring the impact of the COVID-19 pandemic on learning and teaching (17, 18, 22, 23), less is known about individual differences in adaptability to remote education by both students and educators in response to the pandemic. The COVID-19 pandemic is still unresolved, and there is uncertainty about when and how this can be resolved and what the future will be like post-pandemic. Given that adaptability is central to coping with, and problem solving in, new and uncertain circumstances (1, 2), understanding students' and educators' adaptability to the remote education during the COVID-19 pandemic may provide important insights into current and future complex challenges of health professions education. This study aims to explore healthcare students' and educators' adaptability experiences to remote education during the pandemic.

## Methods

### Design

There is complexity of teaching-learning programs and multiple interactions involved in health professions education. This often requires various forms of data to make sense of health education research problems (24). Aligning with this, a mixed-methods design was adopted to facilitate our understanding of the various elements and factors that influence students' and educators' experience of adapting to remote education.

The study reported is part of a larger project which evaluates longitudinally the impact of the change to teaching and learning approaches in selective health disciplines, including remote education and changes to work-integrated learning, during the COVID-19 pandemic (25). This study was underpinned by pragmatism, which suggests pluralistic approaches to address research questions (26, 27). Pragmatism acknowledges both singular and multiple realities, and views knowledge being both constructed as well as based on the reality we are in and interact with (27). We come to know reality using both objective and subjective evidence. Aligning with these views, a convergent mixed-method design was adopted to utilize the power of quantitative and qualitative methods for answering the research questions (26, 28). Quantitative data were collected *via* online surveys of students and educators to capture their perceived adaptation to remote education and the challenges encountered in the process. In addition, group and individual interviews were conducted with selected students and educators to gain a deeper understanding of their experiences. The discussion then integrated these two types of data.

This research was approved by the Monash University Human Research Ethics Committee (Approval number: 24300) and informed consent was provided by all participants. Data reported in this paper were collected between May and August in 2020.

### Study Setting and Participants

Participation was sought from the health faculty at Monash University—an Australian research-intensive university, which has 12 health professions and four health science courses. Disciplines were chosen purposively based on being the largest undergraduate courses in the faculty and included health profession and health science courses (pathways to post-graduate health professions degrees). Courses included medicine, nursing/midwifery, physiotherapy, health science/public health, and biomedical science. The study sample included students and educators of these selected disciplines.

Students from all year levels of these disciplines were invited using an announcement on the learning management system (Moodle) whereas educators were invited *via* email to complete the online survey, *via* Qualtrics. A subsection of the survey respondents voluntarily participated in the second phase of the study—group interviews for students and individual interviews for educators. Seven group interviews with students and 17 individual interviews with educators were conducted.

### Data Collection

#### Phase 1

Separate surveys were administered for student and educator participants at the end of the first semester in 2020 (May–June). The surveys were developed by the researchers and refined through discussion in several rounds of team meeting. Prior to administering, the surveys were piloted with ten students and four educators. Both surveys asked some similar demographic questions (e.g., gender, and discipline). The student survey further asked about year of study, student status (local/domestic) and first language, while the educator survey asked about their academic level. The surveys also asked participants whether they have any experience of remote education prior to the COVID-19 pandemic. We asked participants to rate, on 5-point scales, how prepared and interested they viewed themselves for remote education. For preparedness, the scale ranged from 1 = not all prepared to 5 = extremely prepared, whereas for interest, the scale ranged from 1 = not all interested to 5 = extremely interested.

Both the student and educator surveys included a validated nine-item Adaptability Scale (2) to measure participants' adaptability. When responding to the items, participants were asked to consider their experience of remote education during the pandemic. The items of the Adaptability Scale asked them how constructively they could respond to new, changing, and/or uncertain circumstances, conditions and situations (e.g., I am able to think through a number of possible options to assist me in a new situation). Participants responded to items on a 5-point scale of 1 = strongly disagree to 5 = strongly agree. Additionally, participants were asked about their perceived effectiveness in adapting to changes related to remote education on a 5-point scale, ranging from 1 = not effectively at all to 5 = extremely effectively. It was anticipated that participants with higher level of adaptability, as measured by the Adaptability Scale, would report higher effectiveness in adapting to changes related to remote education.

**Figure 1:**
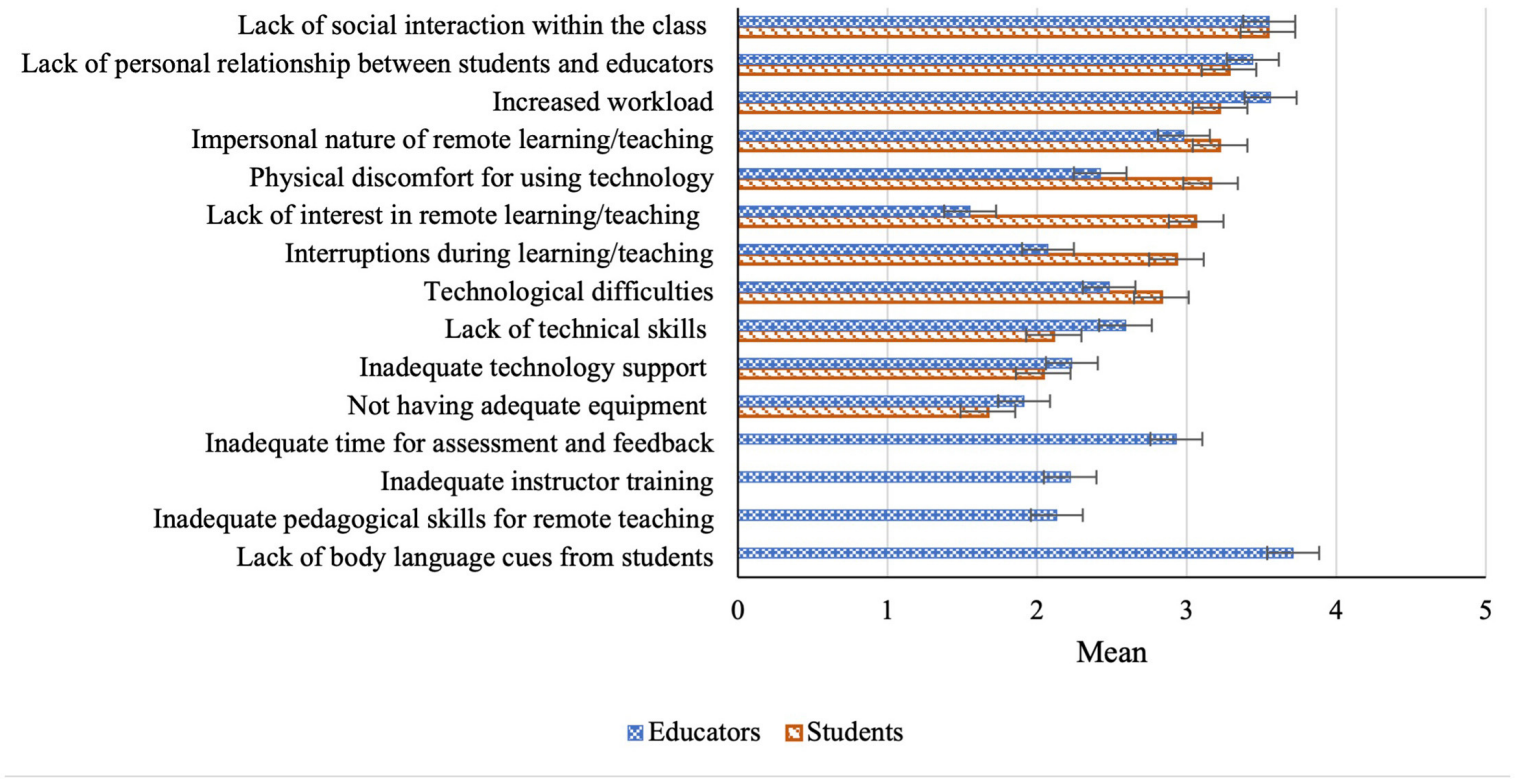
Students' and educators' challenges to adapting to remote education. 1 = Not at all a challenge, 2 = A minor challenge, 3 = A moderate challenge, 4 = Quite a challenge, 5 = A serious challenge.

Drawing on previous studies [e.g., (29, 30)] we compiled factors which might have challenged students and educators to adapt to remote education. The factors represented interpersonal types (e.g., lack of personal relationship), technology-related (e.g., inadequate technology support), and cost–benefit types (e.g., incremental change in workload burden). Both the student and educator surveys included 11 factors in common, wherein the educator survey had four additional factors (see Figure 1). These factors were: inadequate time for assessment and feedback, inadequate instructor training, inadequate pedagogical skills for remote teaching, and lack of body language cues from students. All respondents were asked to indicate the extent to which each factor challenged them in adapting to remote education on a 5-point scale of 1 = not at all a challenge to 5 = a serious challenge. The surveys also collected students' and educators' expressions of interest in participating in the follow-up qualitative phase. A copy of the surveys is available on request.

#### Phase 2

Phase 2 data collection (i.e., interviews) was conducted between June and August in 2020. Group interviews were used for students to stimulate discussion and enable debate in order to understand their views of remote learning experiences (31). For educators, we wanted to capture individual perspectives of their experiences, best facilitated through in-depth interviews, rather than focus group discussion. Also, based on our previous experiences working with busy educators, we perceived that scheduling group interviews might be challenging.

Semi-structured interview protocols were used with necessary probes and prompts allowing the interviewer to explore participants' answers to gain deeper insights and seek clarification (32). The protocols commenced with a general question “what was your experience of learning (or teaching) in this past semester”, which generated intensive discussion about their adaptation to remote education and the associated challenges encountered. The protocols were developed by the research team after the initial analysis of the survey data to identify issues which warranted more exploration. For example, the surveys identified the lack of social learning as a major challenge of remote education for both students and educators. Reflecting on this, we included questions, e.g., “what role did your peers play in your learning in the past semester?” in the students' protocol, wherein the educators' protocol included, “why do you think the majority of educators are concerned about lack of social learning and what may be its impact?”. In the group interview, we also facilitated as much discussion between participants as possible, using questions such as: “Did anyone else have a similar/different experience?”

All group and individual interviews were conducted virtually using Zoom (Zoom Video Communications, San Jose, CA, USA), which were recorded and transcribed for analysis. A total of seven group interviews were conducted with 26 students and 17 educators participated in individual interviews. Student interviews lasted between 44 and 74 min (mean 48 min, total 5 h 33 min) and the educator interviews lasted between 19 and 63 min (mean 43 min, total 12 h). Sufficient information power (33) to analyse and interpret findings was assisted by our focused aim (i.e. stakeholders' experiences of the adapting to remote education), our tight sample specificity (i.e., student and educator stakeholders), the high-quality dialogue in the interviews, and our team-based thematic analysis strategy (34).

### Data Analysis

SPSS (version 27, IBM Corp, Armonk, NY, USA) was used for statistical analysis of the Phase 1 survey data. After screening and cleaning of the data, descriptive statistics for demographic data were summarized using mean, standard deviation for continuous data (e.g., age), whereas, frequencies and percentages were calculated for categorical data (e.g., gender, year of study). Mean and standard deviation were computed for Likert scale data. The relationship between perceived effectiveness to adapting to remote education and adaptability score was analyzed using Pearson correlation coefficient. Significance of differences between groups was evaluated using independent-samples *t*-test for two groups (e.g., comparing adaptability scores between male and female) or one-way between group *ANOVA* for more than two groups (e.g., comparing adaptability scores among the disciplinary background). *P*-value of <0·05 was set as the threshold for statistical significance.

NVivo (version 12; QSR International, Melbourne, Australia) was used to analyze the interview data. Digitally recorded interviews were first transcribed, and then each was scrutinized by simultaneously reading them and listening to their recordings. Four authors (MS, KL, AK, and CP) were involved in the analysis using Ritchie and Spencer's (34) five-stage framework analysis. Using this approach, we first developed a coding framework with students' data and then adapted this for educators' data. The first stage involved a sample of student transcripts being analyzed and initial codes were generated individually across them. The second stage involved discussion in several rounds of team meetings based on which a coding framework was developed. We used the negotiated agreement process to resolve any disagreement and to establish the rigor of data analysis (35). This negotiated agreement process involved us coding the data independently and then regularly meeting to compare, contrast, and come to an agreement about our interpretations. This process was iterative until the final coding framework was agreed on. The coding framework detailed codes, definition/description together with illustrative quotes. The third stage involved each transcript being coded using the coding framework in NVivo. The coding framework was modified as required during this indexing process to complete all interview coding. The fourth stage involved identifying patterns in the data such as the similarities and differences between student and educator perspectives. The final stage involved ongoing discussion among the analysis team to compare, contrast and negotiate our interpretations of each theme and sub-theme and discuss the interpretations of the findings in light of the research literature. The entire process helped to maximize the credibility of the analysis and enhance the rigor of the study.

### Team Reflexivity

Our team of eight was diverse in terms of our research experience and orientations with qualitative and quantitative methodologies, disciplinary backgrounds, and demographics (e.g., age, gender). We had representatives from each target discipline (i.e., medicine, nursing, physiotherapy, biomedical science and health sciences). We completed a team reflexivity exercise (36) at the beginning of the project. This provided us with a valuable opportunity to understand each other's perspectives and served to surface our backgrounds and experiences, and thus potential influences over data collection and analysis. Diversity within our team supported more rigorous data interpretation with team members contributing different perspectives and insights into the data analysis and reporting.

## Results

In this section, we report results on quantitative and qualitative data separately. The results are then examined together through integration in the discussion section.

### Participant Demographics

A total of 476 students out of 717 who opened the Qualtrics link voluntarily completed the survey (completion rate 66%). Ninety-five educators out of 137 opening the Qualtrics link voluntarily completed the survey (completion rate 69%). Incomplete surveys were not included in the analysis. Students' mean age was 21.3 years (*SD* = 4.3) and educators' 47 years (*SD* = 11.6). First year students had the highest representation (29%) followed by third year (27%). The majority of the participants were female in both student (75%) and educator (76%) groups. Medicine students had the highest representation (36%) followed by biomedical science (27%), whereas the highest number of educators represented biomedical sciences (31%) followed by nursing/midwifery (26%). The majority of the student participants were local/domestic students (82%) with English as their first language (79%).

A total of 26 students participated across seven focus group interviews. Focus groups were heterogeneous, i.e. participants represented different disciplines and year levels. Female (72%), local students (68%) were the majority with half of the students representing medicine. First year students had the highest representation (36%) followed by fourth year (27%). Of the 17 individual educator interviews, the highest representation was from nursing (*n* = 5). Medicine, physiotherapy, biomedical science, and health science had equal participation (*n* = 3 each).

### Phase 1 Results

#### Previous Experience of Remote Education

Two-thirds of the students (66%) reported that they did not have any experience of remote education prior to the COVID-19 pandemic. Of the educators, almost half of them (49%) had previous experience providing remote education.

#### Adaptability to, Preparedness for, and Interest in, Remote Education

For the Adaptability Scale, Cronbach's alpha for student and educator population calculated as 0.88 and 0.87, respectively, indicating the scale has high internal consistency (37) for our sample. So, scores as measured by the Adaptability Scale can be considered reliable.

As Table 1 presents, the Adaptability Scale yielded a higher score for educators, indicating their higher adaptability to remote education than students. Neither students nor academics seemed prepared for remote education, leaving the mean score <3 for both groups. Students' interest in remote education also seemed relatively low, whereas academics were significantly more interested in remote education.

**Table 1:** Students' and educators' adaptability and perceived effectiveness in adapting to, preparedness for, and interest in, remote education.

	**Adaptability**			**Perceived effectiveness in adapting**			**Preparedness**			**Interest**		
	**Mean**	**SD**	***t***	**Mean**	**SD**	***t***	**Mean**	**SD**	***t***	**Mean**	**SD**	***t***
Students (*n* = 476)	3.57	0.61	9.22^**^	3.18	1.01	8.89^**^	2.38	1.03	2.34^*^	2.22	0.67	16.71^*^
Educators (*n* = 95)	4.19	0.50		3.98	0.74		2.65	1.06		3.68	1.19	

* *Significant at the 0.05 level*;

** *Significant at the 0.01 level*.

A Pearson correlation test indicated a strongly positive correlation between participants' adaptability scores and their perceived effectiveness of adapting to remote education (students: *r* = 0.41, *p* < 0.01; educators: *r* = 0.59, *p* < 0.01). This result aligns with our anticipation that participants with higher level of adaptability would report higher effectiveness in adapting to changes related to remote education. Similarly, strongly positive correlations were found between adaptability scores and their preparedness (students: *r* = 0.35, *p* < 0.01; educators: *r* = 0.22, *p* < 0.05) and interest (students: *r* = 0.14, *p* < 0.01; educators: *r* = 0.26, *p* < 0.05) in remote education.

The sub-group analysis for the adaptability score was done using independent samples *t*-test (for two groups) or one-way between group ANOVA (for multiple groups). For the student cohort, their adaptability to remote education did not differ statistically for gender, discipline, year level, student status and any previous experience of remote education. Similarly, educators' adaptability to remote education did not differ statistically for gender, discipline, academic level and any previous experience of remote education.

#### Challenges to Adapting to Remote Education

Figure 1 illustrates the extent to which students and educators perceived the factors as challenges to adapt to remote education, based on the mean scores calculated. On average, all of the factors received a mean score of <4, indicating that those were perceived as moderately to quite challenging by the participants. Factors of interpersonal types (e.g., lack of social interactions and lack of personal relationship) and cost–benefit types (e.g., increased workload) were the bigger challenges for both students and educators. In comparison, lack of interest in remote education, physical discomfort and being interrupted were more challenging for students than educators. It appears from Figure 1 that, of the 15 factors for educators, participants found the lack of body language cues from students as the most challenging factor.

### Phase 2 Results

Analysis of qualitative data identified common themes representing the educational experiences students and educators had while adapting to remote education. The themes included: social learning, teaching philosophy, technical and non-technical skills, and supporting adaptability.

#### Social Learning

The major concern educators and students expressed in adapting to online learning was limited social learning that occurred during remote education. They observed the loss of body language cues (Table 2, quote 1), artificial and impersonal interactions (Table 2, quotes 2–3), limited conversation (Table 2, quote 4), weaker rapport (Table 2, quote 5), and little peer to peer debate and discussion that diminished the value of deep learning (Table 2, quotes 6). Educators were concerned about the impact of limited peer learning on student ability to discuss and critically evaluate learning content with their peers. They reported that students take educators' words as absolute truth but are more likely to critically evaluate what their peers say (Table 2, quote 7).

**Table 2:** Illustrative quotations for social learning.

1.	I guess, face to face contact, even thinking back to lectures, being able to read body language, things like that being face to face, having bodies in a room. It is I guess, more difficult to keep up with students who maybe you are a little be concerned about, and that comes back to the body language, how they're interacting with their teammates and things like that. I felt like that was, I was a bit more removed from that. (EI2)
2.	I find there a little bit of awkwardness. You know, in zoom chat, someone wants to go say something, it's like, No, you go, No, you go, you go. I think that it's made the interaction a bit more sort of stilted. I don't think it's quite as natural as it would be if you're in person. Yeah, I think it's definitely been a I think it's been a factor. (SFG7)
3.	I think from a teacher's perspective, I find it very difficult because you can't, you're not getting any feedback from students. And if they're not turning their cameras on or they're not engaging, it's very hard. So you're chatting to this screen and you're not getting anything back. (EI1)
4.	Probably I just think we lack the connection and the ability to ask questions that we do in the pracs, where we can just stop and talk to you. (SFG1)
5.	I don't have the same rapport the students that would normally have and in saying that I feel like and I'm not trying to blow air and trumpet but I feel like our students feel like they've got struck a strong connection to us and they might have with other educators. But yeah, I don't feel like we've got that rapport that we would normally have. (EI4)
6.	Discussions with peers support deep learning, not much has really stuck because you know, having those discussions with your friends about things is not happening. (SFG4)
7.	Students are much more likely to critically evaluate what their peers say than what we say if we say something, they'll hear it the way they think they've heard it. And they will hold that misconception for years to come. Because they heard it from us, but really they didn't hear it from us, they heard us say one thing in their head that they thought they heard. But when their peers say it, they're more likely to critically evaluate it, they're more likely to really analyse and work out how that fits in with our own understanding. We've lost that ability this year. (EI9)
8.	I'm in a breakout room, I don't know what's happening in the other four breakout rooms, because I can't see them. Whereas, I could see it on a table before. And I could go well, I know that there's no other hands up, I know, I can see everybody else is working, I can keep an eye on the rest of the class. But I know that you're having trouble. So I'm going to sit here and try and get you to think it through rather than to tell you why. Now I'm like, okay, I've spent 5 min in this room already. I need to go make sure the other rooms are okay. You know, I don't know if they finished or not finished or whatever. (EI9)
9.	You know, normally you look in a classroom without being intrusive, but then when you go into a breakout room, everything stops or the whole dynamic changes. Either they notice you and the whole dynamic changes, or they don't notice you and then I feel like a stalker. (EI9)
10.	I think that motivation is the largest role that my peers play in my learning, being able to discuss content with them whether it's incidental in terms of on the wards or just *via* studying together or, more formally in a face to face setting and PBL. That's non-existent now and I find with the online modules, lacking the social element of learning impacted my engagement in learning. (SFG3)
11.	I think so often, I almost forget that I'm doing a course and there's other people also doing it, because it's essentially kind of just me in my house studying. Um, so I think it's really that social aspect that I'm missing, which kind of pulls everything and makes the whole process more enjoyable, and kind of exciting. (SFG5)
12.	Cannot make new friends, because it was very difficult to do that in the zoom environment. (SFG4)
13.	Before we go by just the incidental kind of social things like people you wouldn't necessarily, you know, send a message on Facebook, but you'd say hi to them in the corner, and you'd have a bit of a chat and stuff like that. That's something I've definitely missed. (SFG4)
14.	all the informal learning that happens between students in the corridors, meeting someone in the toilet cubicle, you know, like when you're washing your hands and having a chat at the library, walking down to get a coffee, you know, not just the formal classroom workshops, but all of that stuff is just pivotal to being socialized into University, and ask those trivial questions and all of that. (EI14)

*EI, educator interview; SFG, student focus group*.

In response, educators attempted to replicate group discussion using breakout rooms that would usually happen around tables in a face-to-face environment. However, they struggled to keep track of the discussions being had in each group due to the inability to physically visualize the classroom (Table 2, quote 8). Also, they noticed that their presence in a breakout room affected the conversation dynamics and the level of engagement generally developed in a face-to-face classroom (Table 2, quote 9).

Students appreciated how important their peer's role was on their learning motivation and commented on how limited peer interactions impacted their engagement in learning (Table 2, quote 10). As a result of limited peer support, learning experiences for students became less enjoyable and boring (Table 2, quote 11).

Students struggled to make new friends online (Table 2, quote 12). They missed out on the incidental interaction opportunities to meet people that would happen in a face-to-face environment (Table 2, quote 13). This affected their ability to socialize with their peers, and extend the learning and conversation from the confines of the classroom to the wider space and University campus (Table 2, quote 14).

#### Teaching Philosophy

Adapting to remote education required educators to change their teaching philosophy. They reported the loss of being organic and responsive in teaching remotely that is naturally fostered in the in-person environment (Table 3, quote 1). This prompted them to shift to a more didactic pedagogy, given their limited knowledge of making online lessons engaging (Table 3, quote 2). Educators also recognized that students took on a more passive stance in learning, requiring more prompting from teachers (Table 3, quote 3). Students found the didactic approach less engaging and less efficient for learning (Table 3, quote 4).

**Table 3:** Illustrative quotations for teaching philosophy.

1.	I'm very organic and bit more responsive in my teaching. And so I would take cues when I was teaching face to face to know which direction or I'd find those teachable moments where the gold is, you know, where you feel that buzz in the room that lights have gone on their heads. And, and I didn't have any of that. (EI4)
2.	I don't talk very much, they do all the talking with each other … that's sort of the basis of my teaching philosophy. I had to sort of revert back to didactic teaching because I didn't know how to really engage them in that peer to peer learning online in such a short period of time. (EI14)
3.	I'm becoming one of those old fashioned teachers who is more likely to go in there and say, Well, this is what the answer is, even if I do probe them for a bit of understanding, I'm still much more likely than I would have been in previous years to be able to, you know, to feel the pressure of time, perhaps and then maybe probe them a little bit, but kind of give in to them and give them an answer. Whereas, in the past I would say I would sit and problem solve with them. (EI9)
4.	Didactic teaching is already difficult to engage with at times, let alone on Zoom, and months of it was not an ideal way to learn. (SFG3)
5.	Teaching this way has been a challenge at times to stay engaged, to be honest. The inherent interest is still there. But I think this is just a consequence of being isolated and not having the change of scene and yeah, being alone. I think the engagement that it's, it has flagged at times, and you kind of do feel like I just want to hide a little bit from students. (EI7)
6.	I felt really insecure about my teaching online but I just not as I said, I'm not as good a teacher now, as when I was in the classroom. (EI2)

Educators reported the shift to transmissive pedagogy affected their own levels of engagement, causing feelings of exhaustion and disconnect with students compounded by social isolation (Table 3, quote 5). This contrasted starkly with the energy naturally fostered in face-to-face learning environments, despite retaining the synchronous learning environment and interacting with students remotely. Additionally, this shift prompted educators to feel less competent in online teaching (Table 3, quote 6).

#### Technical and Non-technical Skills

Adapting teaching that previously was heavily centered on clinical placement and laboratory/practical classes into an online learning environment was reported as challenging. Educators and students noted the adverse impact that remote education had on students' technical skills development. This resulted in reduced student ability to effectively develop and practice these skills in real-time with their peers and teachers, and there was also a loss of the reasoning and critical analysis that is generally garnered from the physical involvement with a practical or a laboratory-based class (Table 4, quote 1). Educators noticed the shift to theoretical learning without applying this theory in a real-world, practical context (Table 4, quote 2). Similarly, students viewed that a lack of hands-on learning opportunities would result in limited clinical and practical skills (Table 4, quote 3).

**Table 4:** Illustrative quotations for technical and non-technical skills.

1.	I was teaching a very lab heavy, practical, focused unit, and that we basically had to set all of the technical side of the unit aside. So I think that's a big hole for the students that ordinarily were in a unit that really teaches them a lot of the fundamentals that they take on to other units, and throughout their degree. And the students obviously didn't gain any of those technical skills this semester. (EI6)
2.	The immediate impact is a complete disruption to the manner in which they learn and the skills that they learn. They are really forced into much more theory, and relying on to some degree of roleplay and new technologies, which they've not done before and a lot of them struggle a bit with that. (EI2)
3.	They did provide some videos that about how to, like provide a vital sign or measure blood pressure, actually, my brain was in but my hands are not on it. It's hard to transfer the knowledge from my brain to my hand. (SFG2)
4.	Online learning doesn't encourage questions and doesn't encourage critical thinking when students aren't surrounded by other people, and less team working and communication. So you miss the soft skills. (EI3)
5.	Perhaps a growing cohort of our students who come from culturally linguistically diverse backgrounds that don't have that same basis and don't have that same exposure to Australian culture and our sort of local cultural and communication practices. And I think that will impact quite profoundly on them. (EI15)

Not only was the development of technical skills impacted, but educators also perceived the change in the physical learning environment to hinder students' abilities to develop non-technical skills (i.e., transferable skills, e.g., communication, teamwork and critical thinking) necessary for entering the workforce (Table 4, quote 4). Particularly, they perceived that students from culturally and linguistically diverse backgrounds would be disadvantaged in terms of limited key competencies as they missed the opportunity to interact with local students (Table 4, quote 5).

#### Supporting Adaptability

Many students viewed health professions as a rewarding career and felt that this view helped them keep focused on remote learning (Table 5, quote 1). They considered that they were part of a community going through the challenges caused by remote learning. Together with being resilient and continuing learning, this view supported their adaption (Table 5, quote 2). Students also recognized that how well they adapted to remote education did not necessarily indicate their liking for it but their acceptance of it for the time (Table 5, quote 3). For educators, having an intrinsic interest and passion in teaching and resource sharing and supporting other educators helped facilitate student learning in a remote environment (Table 5, quotes 4–5). They created a supportive structure for students through frequent check-ins and pastoral care (Table 5, quote 6). They were compassionate to student needs and ensured flexibility when required, for example, in assignment extensions (Table 5, quote 7). Both students and educators viewed the critical importance of a positive attitude to change, and viewed remote education as an opportunity to learn and grow to adapt to future changes (Table 5, quotes 8–9).

**Table 5:** Illustrative quotations for supporting adaptability.

1.	For all of us, we're doing courses that especially now during like a pandemic, we realize, how important they are, we look at, like the doctors, the researchers, the nurses, like all those frontline kind of essential workers as heroes. So I think, seeing that you can also be part of it and essentially contribute to that is also something really special, rewarding (SFG5)
2.	All students are having, yeah, going to the same situation like you. … I think having that resilience to just keep pushing, keep learning, keep safe to make sure that you stay on top of those things really is important. (SFG4)
3.	I felt like I adapted quite quickly to online learning. That doesn't mean to say that I like it or I'm interested in it. It's just something that I've accepted now. (SFG3)
4.	If you're someone who I think is intrinsically, you want to see students learn, you want to challenge yourself in the way that you deliver content and get students involved and interactive. … I think the intrinsic interest is really important. (EI7)
5.	I think there's been a lot of resource sharing. … I think that's been a definite positive people being willing to say, yeah, sure, have a look at my Moodle site, take what you need. (EI6)
6.	I was a bit more plugged in, in terms of monitoring them and touching base, a lot of more unit announcements coming out every week just to check in and that pastoral care element. (EI3)
7.	There was a lot of extensions given for assessments. And marking it allowing for this particular, you know, pandemic and the anxiety that that would bring and all of those things. (EI14)
8.	Change happens all the time and how to adapt change in what the positives might be of the change. And so yeah, it's a willingness to adapt to change … is such an integral part of managing this whole situation in all aspects of our lives, not just in teaching. (EI16)
9.	When started [the semester], no one would have thought they've been doing the whole course online. We've all had to adapt to all the changes, so we are more prepared for any changes. (SFG5)

## Discussion

This study aimed to explore healthcare students' and educators' experiences while adapting to remote education. Integrating quantitative measurement of students' and educators' adaptability and qualitative exploration of their adapting experiences together showed that students were less adaptable than educators, and while remote learning was less appealing, some adapted well to it. Reduced opportunities for social learning, combined with a move to transmissive pedagogy and lack of clinical and transferable skills development, characterized participants' adapting experiences. Despite this, students and educators utilized adaptive strategies to address the shift to remote learning. Implications of these key findings will now be explored in light of the literature.

Adapting to the lack of social learning was found to be a challenge for both students and educators. Additionally, for educators, the most challenging factor was the lack of student body language cues—an aspect of social learning (16, 38). Educators described initiatives that attempted to maintain social learning using digital platforms. Other evidence has also suggested that educators worked harder to manage chatrooms and collect non-verbal cues from students that added to their workload and influenced their adaptability (38). The limited peer to peer interactions was reported to promote surface learning and student reliance on teachers. Others have highlighted the critical role of peer to peer interactions for promoting deep learning (39).

Adapting to remote learning hindered the development of technical (i.e., clinical) and non-technical (i.e., transferable) skills of students given their limited exposure to rich learning experiences encompassing authentic problem solving in clinical settings and collaborative learning with peers. Both clinical and transferable skills are essential for students' preparedness for practice or employability post-graduation (40). In addition, transferable skills (e.g., collaboration, communication and cultural competence) contribute to learning in a multicultural education environment comprising diverse student cohorts (41). Our finding, therefore, questions the fitness of remote education to prepare future healthcare graduates.

A striking finding suggested from our qualitative data was that educators adapted by shifting to transmissive pedagogical approaches. This contradicts the advocated pedagogical approaches for effective student learning, especially in remote education settings (16, 42). As Rapanta et al. suggested, in remote educational settings, the educators' role is more focused on facilitation (than direct teaching), while students take ownership of their learning. However, educators in our research focused on delivering content using didactic pedagogy, whereas students took a more passive role. Nonetheless, being a passive learner was not appreciated by our student participants, as aligned with recent studies (43–45). Data suggested educators' awareness of the notion of ‘teacher presence' is significant for online learning (46), yet limited knowledge of how to engage students more effectively in remote settings, coupled with lacked time to make a more planned approach, attributed to the change in their teaching philosophy and disconcerting adaptability experience to remote education. This finding suggests the need for further professional learning opportunities for educators to design student-centered and engaging online learning episodes for students.

Strong career aspiration and positive attitudes supported students in adapting to remote education, whereas educators recognized the role of passion and intrinsic interest in teaching, collaborative and supportive network, and being flexible and compassionate to student need in their adaptation mechanism. Additionally, a willingness to adapt to changes facilitated the adaptation for both groups of participants to remote education. Aligned with previous research (47), this finding attests to the critical role of adaptability and the significant challenges experienced by students and educators in learning and teaching due to the need to rapidly adjust to the changes and uncertainties caused by the pandemic. Given that educators are better at adapting to remote education than students, their teaching may consider the promotion of adaptability mechanisms so that students are better equipped to adapt to future uncertainties. Since adaptability is a sought after skill for employability or preparedness for practice (6), its promotion may mean that students are graduated with higher adaptability to cope better with uncertainties within the future world of work.

Our study found that participants' adaptability did not differ for demographic factors (e.g., gender, student status, academic level) or whether they had previous experience of remote education. This finding does not align with previous research focusing on school-aged students that reported socio-demographic variables (gender, age, language background) as predictors for adaptability (2). Future research can therefore investigate if there are any specific socio-demographic variables affecting the adaptability of University students and educators. It would also be interesting to see if adaptability is associated with academic achievements or personality variables (e.g., extraversion and openness). Future research can also examine how individual participants interpret adaptability and relate this to their cognitive, affective, and behavioral reactions in novel and uncertain workplace situations.

### Limitations and Strengths

The limitation of this study is the single Australian institution focus limiting the transferability of the findings to other institutions and countries. The lack of student participation in latter years of the courses who were likely to have been able to continue with clinical placements, including tele-health is a limitation. However, the study includes both students and educators—two key stakeholders in education—from various health professional courses, and uses a mixed-methods design with voluminous quantitative and qualitative data, meaning a complete picture of the research problem reported in this paper (28).

## Conclusion

This paper reports healthcare students' and educators' adaptability experience to remote education during the pandemic. We found that whilst students and educators adapted to remote education with varying degree, their description of the remote education experiences can mostly be seen as unsatisfying (e.g., limited social learning, transmissive pedagogical focus, and lack of clinical and transferable skills development). These findings prompted us to question the effectiveness of remote education in preparing healthcare students for practice. The COVID-19 pandemic is not over yet. We further anticipate that remote education is likely to become more commonplace for many courses beyond the pandemic. We argue that online health professions education must be accompanied with adequate face-to-face skill development opportunities, and educators must be supported to design student-centered and engaging online learning. Navigating the challenges associated with different modes of education may support students and educators improve adaptability—an attribute critical to managing future uncertainties.

## Data Availability Statement

The datasets presented in this article are not readily available because the qualitative dataset cannot be shared with anyone beyond the research team according to the approved ethics. Requests to access the datasets should be directed to MS, mahbub.sarkar@monash.edu.

## Ethics Statement

The studies involving human participants were reviewed and approved by Monash University Human Research Ethics Committee. The patients/participants provided their written informed consent to participate in this study.

## Author Contributions

MS, KL, AK, and CP were involved in analyzing and interpreting data. MS drafted the paper. All authors designed the study protocol, secured ethics approval, collected data for the study, and critically reviewed and edited various iterations of the paper. All authors gave their final approval for this version to be published and agreed to be accountable for all aspects of the work.

## Conflict of Interest

The authors declare that the research was conducted in the absence of any commercial or financial relationships that could be construed as a potential conflict of interest.

## Publisher's Note

All claims expressed in this article are solely those of the authors and do not necessarily represent those of their affiliated organizations, or those of the publisher, the editors and the reviewers. Any product that may be evaluated in this article, or claim that may be made by its manufacturer, is not guaranteed or endorsed by the publisher.
